# Diseases of the Stomach and Intestines

**Published:** 1904-12-10

**Authors:** 


### Progress in Medicine and Surgery.

### DISEASES OF THE STOMACH AND INTESTINES.

**Nutrition.**—The effects of a purely proteid diet on graminivorous animals has been studied by Watson,[1] who fed eight fowls on a diet of raw lean meat and water. The observations lasted for 16 months. At the end of six months two were in perfect health, two succumbed to acute nervous disease without gross pathological changes. At the end of 16 months three of the remainder had lost weight and suffered from various nervous and cutaneous symptoms, and the fourth had gained weight and thriven remarkably. No traces of gout or uric acid deposits were found post-mortem in any, but the bird which had gained weight was found to be suffering from tuberculosis. The three which lost weight showed marked hypertrophy of the thyroids and parathyroids, and disappearance of the bone marrow. These changes were not found in the tuberculous fowl, in fact the bone marrow was of a leucoblastic type. The main points of practical interest were the absence of uratic deposits, and the fact that raw meat was capable of producing improvement in a graminiferous animal suffering from tuberculosis—an effect which was not found to occur with cooked meat. The supervention of nervous symptoms suggested the value of milk in the treatment of certain nervous disorders, such as chorea and eclampsia. The general subject of nutrition has been discussed by Stern,[2] who brings forward evidence to show that the majority of persons in ordinary circumstances take more food than is necessary to maintain nitrogenous equilibrium. He states that the average diet for an individual to-day represents 35 to 50 calories for each kilogram of body weight, whereas in his experience 20 calories is often enough to maintain the weight of those taking considerable physical exercise. He points out that coarser sorts of food require more energy to assimilate and that, consequently, there is more waste. He does not think that much difference in kilogram-calories can be demonstrated between what is necessary for a man and what is necessary for a woman. For a normal individual 23 to 28 kilo-calories a day of assimilable food are enough, which is represented by about 30 to 35 kilo-calories of ingested food. It is important before attempting to overfeed a person to determine whether his weight is more than 15 per cent. below normal, and whether the caloric value of his diet is low enough to account for this. Further, the amount of work done must be taken into account, as excessive exertion may underlie the malnutrition, in which case over-feeding by putting an extra tax on the digestive and assimilative processes may lead to a breakdown. Hereditary or congenital anomalies in assimilation or metabolism are responsible for most of the cases of apparent underfeeding, in which persons remain lean in spite of sufficient food and moderate exercise. If such persons are in nitrogenous equilibrium, and no tissue waste is going on, treatment by over-feeding is not indicated. The only conditions in which over-feeding is really useful are those when some organic or functional disorder prevents proper assimilation or complete utilisation of food, and in these this underlying disorder must be remedied before the feeding process is begun. Obesity is held by Williams[3] to be traceable to two main factors—errors in the quantity or quality of the food, and errors in the habits of life which prevent the normal oxidation processes of the body. As examples of persons who commit errors in the quantity of food consumed he instances those who have been athletic in youth and continue to take large meals in later life under very altered circumstances, and those (mainly women) who, under the impression that they require "supporting," take frequent meals of a very nourishing kind. Drinking at meal times also aids excessive feeding, as it enables food to pass more rapidly from the stomach, and bolting of food without sufficient mastication also enables an increased amount of food to be swallowed within a reasonable time. As to quality, the effect of certain systems for curing obesity shows that the restriction of carbohydrates and fats does influence the amount of fatty tissue formed, but it must be remembered that most systems also restrict the proteids, in a manner not without danger. An almost purely proteid diet also throws a serious strain on the excretory organs, and may induce granular nephritis. Deficient oxidation of the ingesta is mainly due to deficient muscular exercise, especially seen in the abdominal muscles, where adipose tissue accordingly accumulates. Deficient brain-work also tends to subnormal oxidation, the general effect of the nervous system on nutrition being a well-recognised fact. The activity of the skin is very essential to proper oxidation of the tissues, as also is the efficient action of the other excretory organs. In treating obesity, regimen at a health resort may remedy errors in the quantity and quality of the diet, while fresh surroundings in a foreign country may stimulate the nervous system and encourage active mental processes. Cold baths and light porous clothing stimulate the activity of the skin, whilst hot baths and over-clothing retard it. Woollen underclothing is especially bad, as the material holds in moisture and envelops the patient in a kind of perpetual vapour bath. The fear of a "chill" is a bugbear to the public and also the profession, and leads to elaborate precautions for excluding draughts, with the result that the organism is shut off from an adequate supply of fresh oxygen, without which no oxidation processes can go on. Foreign mineral waters act on obesity in no specific manner, but merely by increasing elimination by the bowels or skin. Thyroid extract, though in most cases effectual in reducing body weight, is not without serious dangers. It acts by increasing metabolic processes, and is liked by patients owing to the simplicity of the treatment; but it should only be prescribed under very careful supervision, as its consequences may be most disastrous.

[1] Brit. Med. Jour., May 14, 1904, p. 1136. [2] Med. Record May 21, 1904, p. 811. [3] Practitioner, May 1904, p. 718.

*(To be continued.)*

